# Update on Weaning from Veno-Arterial Extracorporeal Membrane Oxygenation

**DOI:** 10.3390/jcm9040992

**Published:** 2020-04-02

**Authors:** Enzo Lüsebrink, Christopher Stremmel, Konstantin Stark, Dominik Joskowiak, Thomas Czermak, Frank Born, Danny Kupka, Clemens Scherer, Mathias Orban, Tobias Petzold, Patrick von Samson-Himmelstjerna, Stefan Kääb, Christian Hagl, Steffen Massberg, Sven Peterss, Martin Orban

**Affiliations:** 1 Intensive Care Unit, Medizinische Klinik und Poliklinik I, Klinikum der Universität München, 81377 Munich, Germany; e.luesebrink@med.uni-muenchen.de (E.L.); christopher.stremmel@med.uni-muenchen.de (C.S.); konstantin.stark@med.uni-muenchen.de (K.S.); thomas.Czermak@med.uni-muenchen.de (T.C.); danny.kupka@med.uni-muenchen.de (D.K.); clemens.scherer@med.uni-muenchen.de (C.S.); mathias.orban@med.uni-muenchen.de (M.O.); tobias.petzold@med.uni-muenchen.de (T.P.); stefan.kaab@med.uni-muenchen.de (S.K.); steffen.massberg@med.uni-muenchen.de (S.M.); 2 DZHK (German Center for Cardiovascular Research), partner site Munich Heart Alliance, Medizinische Klinik und Poliklinik I, Klinikum der Universität München, 81377 Munich, Germany; 3 Department of Cardiac Surgery, University Hospital, LMU Munich, 81377 Munich, Germany; dominik.Joskowiak@med.uni-muenchen.de (D.J.); frank.born@med.uni-muenchen.de (F.B.); Patrick.Samson@med.uni-muenchen.de (P.v.S.-H.); christian.hagl@med.uni-muenchen.de (C.H.); sven.Peterss@med.uni-muenchen.de (S.P.)

**Keywords:** venoarterial extracorporeal membrane oxygenation, weaning, weaning strategy, weaning timing, predictors of successful weaning

## Abstract

Venoarterial extracorporeal membrane oxygenation (VA-ECMO) provides temporary cardiac and respiratory support and has emerged as an established salvage intervention for patients with hemodynamic compromise or shock. It is thereby used as a bridge to recovery, bridge to permanent ventricular assist devices, bridge to transplantation, or bridge to decision. However, weaning from VA-ECMO differs between centers, and information about standardized weaning protocols are rare. Given the high mortality of patients undergoing VA-ECMO treatment, it is all the more important to answer the many questions still remaining unresolved in this field Standardized algorithms are recommended to optimize the weaning process and determine whether the VA-ECMO can be safely removed. Successful weaning as a multifactorial process requires sufficient recovery of myocardial and end-organ function. The patient should be considered hemodynamically stable, although left ventricular function often remains impaired during and after weaning. Echocardiographic and invasive hemodynamic monitoring seem to be indispensable when evaluating biventricular recovery and in determining whether the VA-ECMO can be weaned successfully or not, whereas cardiac biomarkers may not be useful in stratifying those who will recover. This review summarizes the strategies of weaning of VA-ECMO and discusses predictors of successful and poor weaning outcome.

## 1. Introduction

Technological improvements during the last decade have enabled a dramatic increase in the utilization of temporary extracorporeal cardiac and respiratory support systems [1]. While veno-venous approaches only supply respiratory support, veno-arterial extracorporeal membrane oxygenation (VA-ECMO)—also known as extracorporeal life support (ECLS)—provides both cardiac and respiratory compensation. VA-ECMO thereby drains deoxygenated blood from the central venous system and returns oxygenated blood to the arterial system. Today, VA-ECMO has become one of the preferred devices to provide temporary cardiopulmonary support for patients with circulatory failure, with or without concomitant respiratory compromise, in highly specialized centers [1,2]. The main indications are cardiogenic shock, cardiac arrest, refractory ventricular tachycardia, right ventricular (RV) failure during left ventricular assist device (LVAD) support, failure to wean off cardiopulmonary bypass, extended in-hospital resuscitation (extracorporeal cardiopulmonary resuscitation (eCPR)), and prehospital use of VA-ECMO [2,3]. The fundamental principle of VA-ECMO is a bridge to (a) recovery, (b) final decision, (c) durable mechanical circulatory support, or (d) heart replacement therapy (i.e., total artificial heart or heart transplant) [4].

Studies reporting standardized strategies for weaning are limited and only few are supported by valid data from large cohorts [5,6,7,8]. This review will discuss (1) different weaning approaches from VA-ECMO, (2) predictors of successful as well as poor weaning outcome, and (3) central elements of promising weaning strategies from today’s perspective.

## 2. Short-Term Outcome of Patients Receiving Veno-Arterial Extracorporeal Membrane Oxygenation (VA-ECMO)

The Extracorporeal Life Support Organization Registry (ELSO) reported overall survival at hospital discharge of 41% for adult patients on VA-ECMO [9]. In the latter registry, data on outcome are limited to observational studies and vary significantly depending on underlying indication. Outcome seems to be most beneficial in patients requiring VA-ECMO support for either acute severe myocarditis, pulmonary embolism with RV failure, or primary graft failure after cardiac transplant, with reported survival rates at hospital discharge of up to 80% [10,11,12,13]. Survival to discharge rate in patients undergoing VA-ECMO and percutaneous coronary intervention in cardiogenic shock complicating acute myocardial infarction is reported to be up to 70% [14,15]. Additionally, VA-ECMO is increasingly used for eCPR to provide circulatory support in patients who fail to achieve a sustained return of spontaneous circulation (ROSC). In patients undergoing eCPR in the setting of out-of-hospital cardiac arrest, survival to discharge rate is reported to be very low between 8% and 29%. In selected subgroups, higher survival rates have been reported [9,16,17]. Taking groups together, outcome of VA-ECMO support remains unsatisfactory, with in-hospital mortality reaching up to 60% [9,15,18].

Of note, risk scores like the Survival After Veno-arterial-ECMO score (SAVE) or the Prediction of Cardiogenic Shock Outcome for AMI Patients Salvaged by VA-ECMO score (ENCOURAGE) have been developed in order to better evaluate the utility of VA-ECMO support and to improve the decision-making process. These are based on pre-ECMO risk factors independently associated with poor outcomes. The related studies revealed older age, female sex, and higher body mass index as well as markers of illness severity including elevated serum lactate levels, renal, hepatic, or central nervous system dysfunction, longer duration of mechanical ventilation, and reduced prothrombin activity as independent predictors of poor outcome [13,19].

## 3. Weaning Does Not Equal Survival

It is important to distinguish between weaning and survival [20]. The proportion of patients with refractory cardiogenic shock who are successfully weaned from VA-ECMO varies between 31% and 76%, depending on the underlying cause [5,6,7,21,22,23,24]. According to recent definitions, we specified successful weaning from VA-ECMO as not requiring further mechanical circulatory support within the following 30 days after VA-ECMO removal [21,22]. However, this clinician’s perspective is quite subjective and, evidentially, 20% to 65% of patients weaned from VA-ECMO do not survive until hospital discharge due to insufficient myocardial recovery, primary or secondary (multi-)organ failure, neurological damage, and other comorbidities [6,8,22,24,25].

## 4. Factors Associated with Poor Outcome of Weaned Patients

### 4.1. Cardiogenic Shock

Studies examining negative predictors of outcome in weaned patients mostly refer to selected VA-ECMO indications. For cardiogenic shock, the following attributes have been identified as independent risk factors of mortality after weaning: advanced age, previous myocardial infarction, diabetes, renal failure with requirement for continuous renal replacement therapy, high serum butyrylcholinesterase, high serum lactate, low mean arterial pressure (MAP), unsuccessful revascularization in the setting of acute myocardial infarction, prolonged VA-ECMO support, hypoxemia at VA-ECMO weaning, low Glasgow Coma Score, and high Sequential Organ Failure Assessment (SOFA) score [20,23,26,27,28,29]. Additionally, Chommeloux et al. recently showed that inability to rapidly restore microcirculation during the first 24 h, which is severely impaired in patients with refractory cardiogenic shock, is associated with death on VA-ECMO [30].

### 4.2. Postcardiotomy Shock

For patients treated with VA-ECMO for postcardiotomy shock, age, gender (female), diabetes, preoperative renal insufficiency, obesity, serum butyrylcholinesterase, mean lactate concentration, lactate clearance, and logistic EuroSCORE are factors reported to be associated with poor outcome [25,31,32,33].

### 4.3. Extracorporeal Cardiopulmonary Resuscitation

For patients undergoing eCPR history of diabetes, obesity, impaired renal and liver function, high lactate levels, postcardiotomy arrest, cardiopulmonary resuscitation duration, door-to-VA-ECMO implantation time, and high SOFA score were associated with death after weaning [16,17,34,35].

Aissaoui et al. noted that depending on VA-ECMO indication, these predictors reflected severity and progression of multiorgan failure at the time of implantation and should be evaluated prior to the first weaning attempt [21].

## 5. Predictors of Successful Weaning from VA-ECMO

### 5.1. Etiology

Among the etiology and patient characteristics, acute severe myocarditis or primary graft failure after heart transplantation are in favor of improved short-term outcomes. The latter are also independent predictors of survival in the ELSO registry [36,37]. In patients undergoing eCPR, younger age, initial rhythm other than asystole, witnessed arrest, and early recovery of blood pressure were predictors of successful weaning and survival as well as favorable neurologic outcome [17,38].

### 5.2. Pulse

Aissaoui et al. were the first to describe pulse pressure as an important clinical factor associated with weaning success [7] as did Pappalardo et al. and Park et al. in their observational studies [6,39]. The latter identified higher mean pulse pressure during the initial 6 h after VA-ECMO implementation as an independent predictor of successful weaning and survival [39].

### 5.3. Echocardiography

Echocardiographic parameters in patients with VA-ECMO support were early on discussed as possible predictors. High values of aortic velocity-time integral (VTI), left ventricular ejection fraction (LVEF), and lateral mitral annulus peak systolic velocity were associated with successful weaning. Parameters reflecting left ventricular (LV) filling pressures (i.e., mitral E and tissue Doppler imaging Ea velocities) and E/Ea contrary predicted worse outcome [7]. Studies that have systematically investigated the influence of RV echocardiographic parameters for weaning success and patient survival are still very rare, and valid statements seem premature due to missing data. However, based on a small cohort study, Huang et al. recently showed that three-dimensional echocardiography-derived right ventricular ejection fraction (RVEF) > 24.6% was associated with higher weaning success and lower 30-day mortality in patients receiving VA-ECMO [40]. Interestingly, this did not apply to tricuspid annular plane systolic excursion and the severity of atrioventricular valvular regurgitation.

### 5.4. Inotropic Support

The degree of acceptable pharmacological hemodynamic support is debated, but, overall, low levels of catecholamines at the time of weaning, reflecting improved intrinsic myocardial function, are associated with improved outcomes [6].

### 5.5. Biomarkers

One of the first authors who investigated the usefulness of cardiac biomarkers to predict cardiac recovery and weaning success from VA-ECMO were Luyt et al. Their prospective, observational, single-center study included 41 patients with refractory cardiogenic shock receiving VA-ECMO support and considered blood N-terminal fragment of B-type natriuretic peptide, troponin Ic, midregional fragment of the proatrial natriuretic peptide, proadrenomedullin, and copeptin concentrations as the predicting parameter. However, none of these biomarkers or their kinetics during the first week differed between successfully and non-successfully weaned patients [41]. Thus, early measurements of these cardiac biomarkers may not be useful as predictors for weaning success. On the other hand, Li et al. showed that early lactate trend is predictive of successful weaning [33]. They investigated the dynamic behaviors of lactate within 6 h and 12 h after the beginning of VA-ECMO support in a retrospective observational study.

## 6. Basic Requirements for Promising Weaning Attempts

The requirements for promising weaning attempt are still controversial and cannot conclusively be answered, since prospective randomized trials are and will be missing within the next years. Even the potential benefit of VA-ECMO therapy on mortality is without scientific evidence to date, because the first randomized trials have just recently been started (EURO-SHOCK (NCT03813134), ECLS-SHOCK (NCT03637205)). Regardless of the underlying indication for VA-ECMO support, the initial weaning trial should not be attempted too early (i.e., within the first 48 h as consensus). The weaning process per se should only be initiated when the patient has sufficiently recovered from the underlying etiology that made VA-ECMO implantation necessary [42]. At this point, a refined decision-making based on the assessment of reversibility of end-organ damage and patient’s overall prognosis is necessary. A continuation of therapy and weaning should be limited to patients with a fair prognosis [43]. Basically, it is important to have regular multidisciplinary discussions involving intensivist, surgeons, cardiologists, patient, and relatives before and during the whole weaning process. An optimal weaning strategy must be based on the patient’s wishes as well as the individual medical/interventional treatment strategy. For instance, it could be relevant to wait for myocardial recovery a few days more under VA-ECMO support for a patient who is not eligible for heart transplantation or LVAD, when for another one, these two options should be discussed earlier in order to optimize the patient’s outcome.

The duration of VA-ECMO therapy in patients suffering from postcardiotomy shock, severe myocarditis, septic cardiomyopathy, arrhythmia-induced cardiomyopathy, or acute myocardial infarction must be compatible with the myocardial recovery [44,45,46]. In myocardial infarction, hibernating myocardium should be assessed properly and possibly recovered, for example [7,8]. Baseline MAP should be ≥ 60 mmHg in the absence or with low doses of catecholamines, and a pulsatile arterial waveform maintained for at least 24 h before starting weaning [6,7,8,22,46]. Hemodynamic instability, mechanical ventilation at maximum level with no prospect of de-escalation, catecholamines in high doses, or unchanged high volume and blood product requirements to maintain an adequate circulation advocate against a weaning attempt.

Aside from myocardial recovery, end-organ recovery and, especially hepatic function, is crucial. Hepatic function should have recovered before any attempt to wean [21,46,47], otherwise secondary mortality remains significantly high. This is in contrast with renal function. There is an agreement that full recovery is not necessary, but thresholds are not clearly defined [21,22,44,48]. For example, recuperation of an acute tubular necrosis could take weeks, and persistence of anuria does not reflect organ perfusion. In such cases, the weaning process will be supported with ongoing hemofiltration. Furthermore, respiratory function requires adequate recovery. Few useful benchmarks have been established within the last years that have applied to many centers. First, pulmonary function should not be severely compromised and pulmonary edema should be reduced to a minimum. A PaO_2_/FiO_2_ ≥ 200 mmHg, an oxygen fraction delivered by the extracorporeal circuit ≤ 25%, and an oxygen fraction delivered by the ventilator circuit ≤ 60% are reasonable for weaning trials. These measurements should be made with VA-ECMO blood flow at 1.5 L/min and sweep gas flow rate of 1 L/min [7,8,22,42,45,49]. Of note, if the patient suffers from persistent pulmonary compromise but sufficient myocardial recovery could be achieved, switching to a VV-ECMO should be considered.

## 7. Weaning Strategies from VA-ECMO

**Figure 1:**
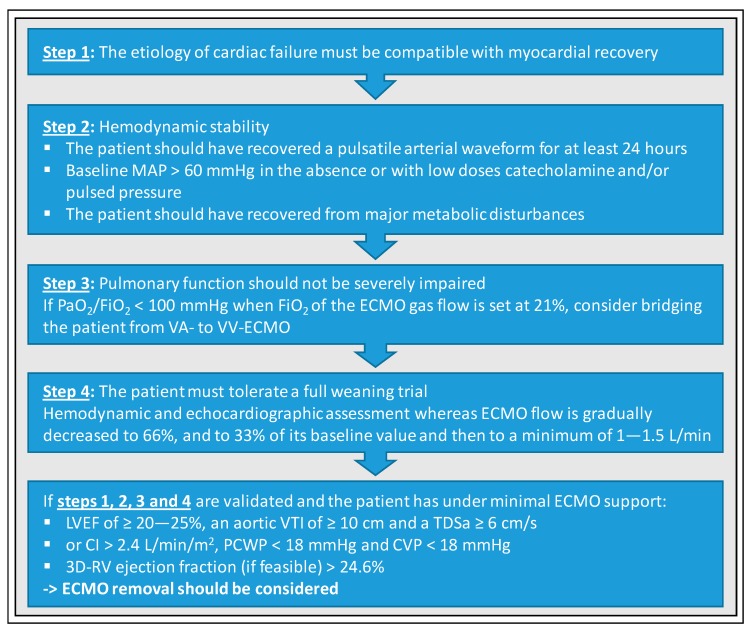
Standardized protocol for weaning from veno-arterial extracorporeal membrane oxygenation (VA-ECMO), according to Aissaoui et al. [21]. MAP, mean arterial pressure; PaO_2,_ Partial pressure of oxygen; FiO_2_, fraction of inspired oxygen; LVEF, left ventricular ejection fraction; VTI, velocity-time integration; TDSa, tissue Doppler lateral mitral annulus peak systolic velocity; CI, cardiac index; PCWP, pulmonary capillary wedge pressure; CVP, central venous pressure; RV, right ventricle.

Different weaning algorithms have been described but data supporting a specific strategy are limited or missing at all. Nevertheless, a standardized algorithmic procedure to optimize the weaning process is indisputable. One of the first weaning algorithms was developed by Aissaoui et al. and is shown in Figure 1 [21].

Many of the requirements for promising weaning attempts, that were above-mentioned, have been incorporated into this algorithm. First, at the beginning of a weaning process, the clinicians should check whether the etiology of cardiac failure is compatible with myocardial recovery. Second, hemodynamic stability must be evaluated (i.e., pulsatile arterial waveform should have recovered for at least 24 h, MAP should be > 60 mmHg, and patient should have recovered from major metabolic disturbances for at least 24 h). Third, pulmonary function should not be severely impaired (i.e., a PaO_2_/FiO_2_ ≥ 200 mmHg). Fourth, the patient must tolerate a full weaning trial and hemodynamic as well as echocardiographic assessments considering LVEF, lateral mitral annulus peak systolic velocity, LV flow (aortic VTI) and RV diameters must be performed whereas VA-ECMO flow is gradually decreased to 66% and to 33% of its baseline value, and then to a minimum of 1–1.5 L/min for at least 15 min. When ECMO flow is reduced, causing an increase in LV preload and a decrease in afterload, behavior of LV and Frank–Starling reserve should be assessed [50]. Aissaoui et al. recommend the use of transthoracic instead of transesophageal echocardiography. If these four steps have been successfully validated, ECMO removal can be considered, if the following requirements are met under minimal VA-ECMO support: LVEF ≥ 20–25%, aortic VTI ≥ 10 cm, and lateral mitral annulus peak systolic velocity ≥ 6 cm/s.

**Figure 2:**
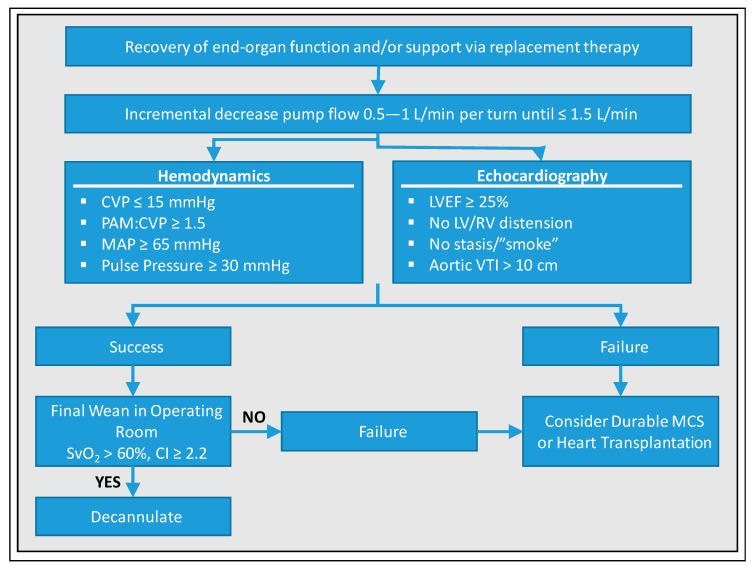
Standardized protocol for weaning from VA-ECMO according to Keebler et al. [18]. CVP, central venous pressure; PAM, pulmonary arterial mean pressure; MAP, mean arterial pressure; LVEF, left ventricular ejection fraction; LV, left ventricle; RV, right ventricle; VTI, velocity-time integral; SvO_2_, mixed venous oxygen saturation; CI, cardiac index; MCS, mechanical circulatory support.

**Figure 3:**
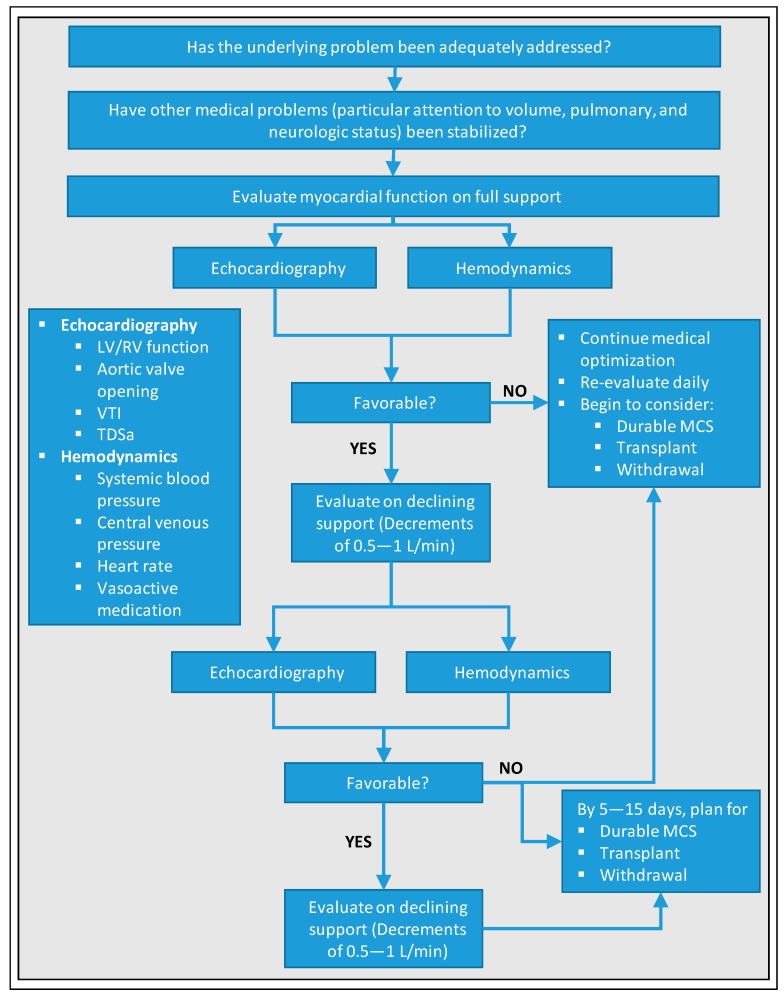
Standardized protocol for weaning from VA-ECMO according to Eckman et al. [10]. LV, left ventricle; RV, right ventricle; VTI, velocity-time integral; TDSa, tissue Doppler lateral mitral annulus peak systolic velocity; MCS, mechanical circulatory support.

Another weaning algorithm was suggested by Keebler et al., which requires a pulmonary artery catheter and is summarized in Figure 2 [18]: In patients deemed ready for weaning (i.e., adequate recovery of end-organ function and/or support via replacement therapy), pump flow should be decreased by 0.5–1 L/min until ≤ 1.5 L/min. This increases preload, allowing the assessment of cardiac recovery. The authors demand following target values based on currently available data for qualifying this step as a success: Central venous pressure (CVP) ≤ 15 mmHg, pulmonary arterial mean pressure (PAM):CVP ≥ 1.5, MAP ≥ 65 mmHg, pulse pressure ≥ 30 mmHg, LVEF ≥ 25%, no relevant LV/RV distension, no stasis/”smoke”, and aortic VTI > 10 cm. If the results are satisfactory, the authors recommend a final weaning with mixed venous oxygen saturation (SvO_2_) > 60% and cardiac index (CI) ≥ 2.2 in the operating room, which allows for controlled decannulation on one hand, and controlled recannulation and reinstitution of VA-ECMO support if necessary, on the other hand. If cardiac recovery cannot be achieved despite medical optimization and recovery of end-organ function, durable mechanical circulatory support should be considered but will be restricted to very few cases. If a fair perspective is missing, withdrawal of support must be discussed. The weaning strategy of Eckman et al. corresponds essentially to that of Keebler et al. and uses the same echocardiographic and hemodynamic parameters, so that a more detailed representation is redundant at this point and reference is made to Figure 3 [10]. Of note, the latter emphasize, particular attention must be given to anticoagulation during weaning such as maintaining therapeutic anticoagulation when flow rates are ≤ 2 L/min, since risk of thrombosis increases with lower circuit flow.

**Figure 4:**
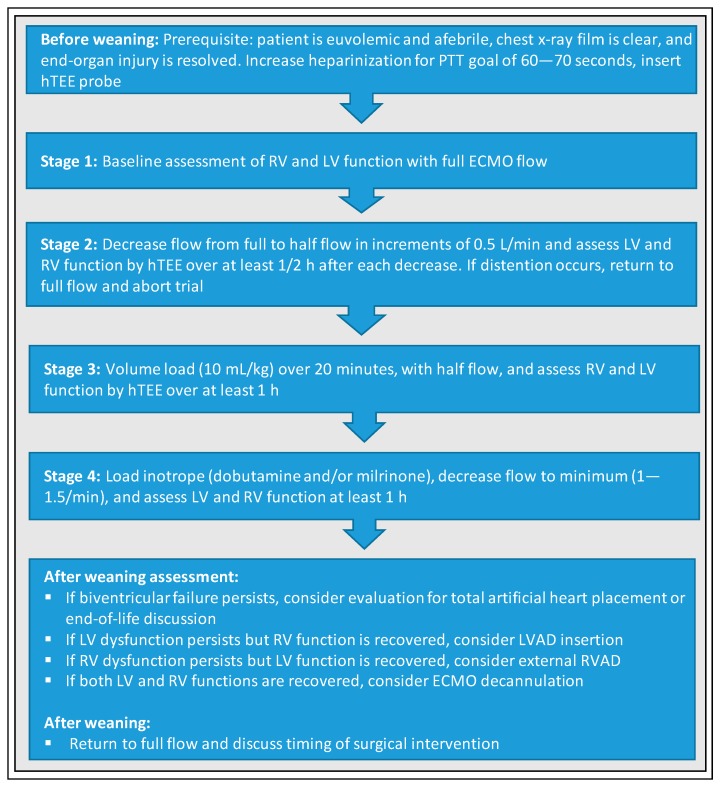
Standardized protocol for weaning from VA-ECMO according to Cavarocchi et al. [5]. PTT, partial thromboplastin time; hTEE, hemodynamic transoesophageal echocardiography; LV, left ventricle; RV, right ventricle; LVAD, left ventricular assist device; RVAD, right ventricular assist device.

Another interesting weaning protocol was developed by Cavarocchi et al. [5] and is based on a miniaturized transesophageal echocardiography probe designed for continuous hemodynamic monitoring, named hemodynamic transesophageal echocardiography (hTEE), and assesses ventricular function and volume status along with hemodynamics during ECMO weaning (Figure 4). Before starting weaning, the patient should be euvolemic and afebrile, chest x-ray film should be clear, and end-organ injury should be resolved. The weaning trial itself consists of four stages. First, baseline LV and RV functions must be assessed on full-flow VA-ECMO support. During the second stage, the ECMO flow is gradually decreased in steps of 0.5 L/min from full to half flow and LV as well as RV function are evaluated by hTEE over at least half an hour after each step. If LV or RV distension occurred, VA-ECMO support is returned to full flow and weaning is stopped. In the third stage, RV and LV function are assessed by hTEE over at least 1 h during elevated preload triggered by volume loading with 5% albumin (10 mL/kg) over 20 min and a reduction of VA-ECMO flow to a minimum rate of 1.2–1.5 L/min. Finally, biventricular function and hemodynamics are evaluated after loading with inotropes (e.g., dobutamine or milrinone) for at least 1 h (a few hours for milrinone) at the minimum flow rate (1–1.5 L/min). Subsequent recommendations depend on the determined LV and RV function. In the case of an appropriate biventricular recovery, patient can be considered for definitive VA-ECMO removal. If LV dysfunction persists, but RV function was recovered or improved with inotropic support, LVAD placement should be considered. In contrast, if RV dysfunction persists, but LV function is recovered, external RVAD placement should be evaluated. Finally, if there is still biventricular dysfunction, repeated assessment, total artificial heart placement, and end-of-life care must be discussed with intensivists, surgeons, cardiologists, and family members. Of note, the authors consciously reject the use of a Swan–Ganz catheter in patients on VA-ECMO support due to safety issues such as migration of the catheter or introduction of air to the ECMO system as well as unreliability of the Swan–Ganz parameters resulting from suction of the venous ECMO cannula.

**Figure 5:**
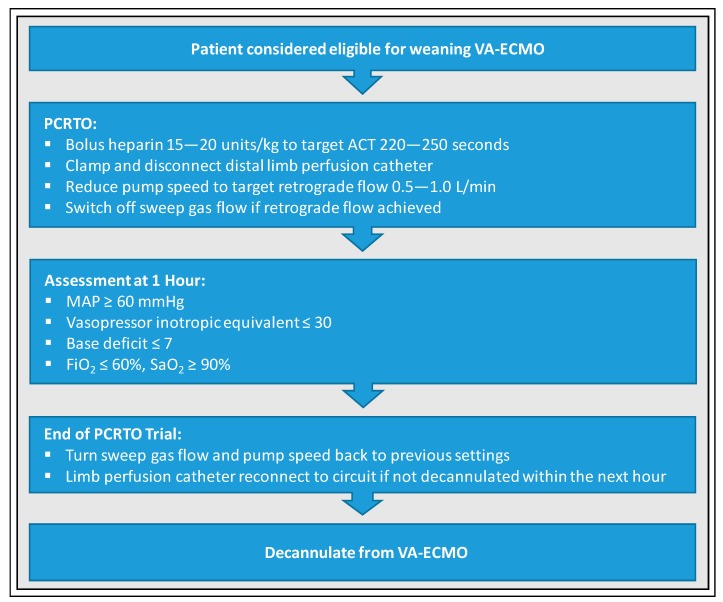
Standardized protocol for weaning from VA-ECMO according to Ling et al. [51]. PCRTO, Pump Controlled Retrograde Trial Off; ACT, activated clotting time; MAP, mean arterial pressure; FiO_2_, fraction of inspired oxygen; SaO_2_, arterial oxygen saturation.

The standardized protocol for weaning of adult patients on VA-ECMO according to Ling et al. [51] was originally based on a technique for ECMO weaning in neonates with respiratory failure reported by Westrope et al. (Figure 5): the Pump-Controlled Retrograde Trial Off (PCRTO) [52]. Pump speed is gradually reduced in a controlled manner until circuit flow becomes retrograde, ensuring adequate RV filling and proper assessment of RV function. The following steps are carried out during PCRTO. First, distal limb perfusion catheter is clamped and disconnected after initial heparin bolus (15–20 units/kg) targeting activated clotting time (ACT) of 220–250 s to minimize clot formation risk under low flow settings. Then, pump speed is reduced until a retrograde flow of 0.5–1.0 L/min is achieved with subsequent turn-off of the sweep gas flow. Since the circuit becomes an arteriovenous shunt, the revolving pump head acts as a resistor, preventing a significant drop in systemic vascular resistance during PCRTO. Patients can be considered ready for decannulation if the following hemodynamic and echocardiographic criteria are met after 1 h: MAP ≥ 60 mmHg, vasopressor inotropic equivalent ≤ 30 (vasopressor inotropic equivalent = dopamine × 1 + dobutamine × 1 + epinephrine × 100 + norepinephrine × 100 + isoproterenol × 100 + levosimendan × 15 (all in μg/kg/min)), base deficit ≤ 7, FiO_2_ ≤ 60%, and SaO_2_ ≥ 90%.

**Figure 6:**
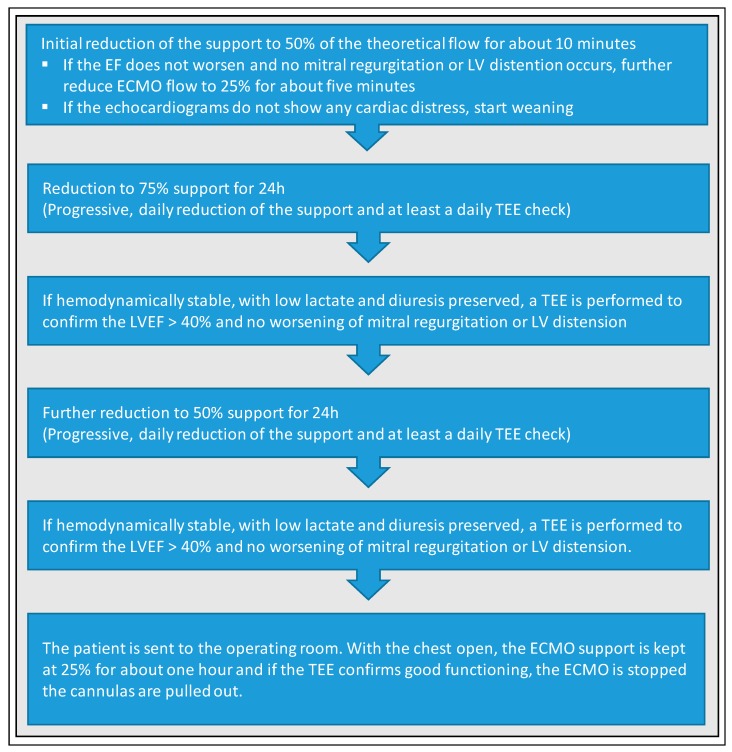
Standardized protocol for weaning from VA-ECMO for graft failure after heart transplantation according to Santise et al. [53]. TEE, transoesophageal echocardiography; LVEF, left ventricular ejection fraction; LV, left ventricle.

Santise et al. developed a special weaning protocol from VA-ECMO for graft failure after heart transplantation (Figure 6) [53]. The patient is considered for weaning trial, if LVEF on full support reaches 40%. Initially, support is reduced to 50% of the theoretical flow for about 10 min. If LVEF does not worsen and no mitral regurgitation or LV distention occurs, ECMO flow is further reduced to 25% for about five minutes. If the echocardiograms do not show any cardiac distress, weaning based on daily reduction of VA-ECMO support and at least one TEE a day can start. After a first reduction to 75% support for 24 h and if patient is considered stable (i.e., low lactate and diuresis preserved, LVEF > 40% and no worsening of mitral regurgitation or LV distension confirmed by TEE), support is further reduced to 50% for 24 h. Again, if the patient is considered stable, the patient is sent to the operating room. Here, the VA-ECMO support is kept at 25% for about one hour and if the TEE confirms good functioning, ECMO is stopped and de-cannulation can be performed.

Finally, in some studies, evidence was found that levosimendan could increase the chances of the success of weaning trials and the integration of levosimendan in weaning strategies was discussed [54,55]. The maximum hemodynamic response of levosimendan as a calcium sensitizer with inotropic and vasodilatory effects is seen 24–48 h after stopping infusion, but its effects can persist for 7–9 days due to active metabolites. Affronti et al. investigated whether the use of levosimendan improved weaning outcomes in six patients suffering from cardiogenic shock and treated with VA-ECMO who received levosimendan 24 h before planned weaning. In their case series, pretreatment with levosimendan seemed to facilitate weaning from VA-ECMO, reducing the need for high-dose inotropes [54]. Distelmaier et al. could also show beneficial effects of levosimendan on survival in patients undergoing VA-ECMO therapy after cardiovascular surgery. Here, patients in the treatment group received levosimendan within the first 24 h after initiation of VA-ECMO therapy [55]. However, these studies are based on small patient cohorts and prospective randomized trials are needed to further investigate the role of levosimendan in ECMO therapy.

## 8. VA-ECMO Weaning—University of Munich, Cardiologic ICU, Weaning Protocol

**Figure 7:**
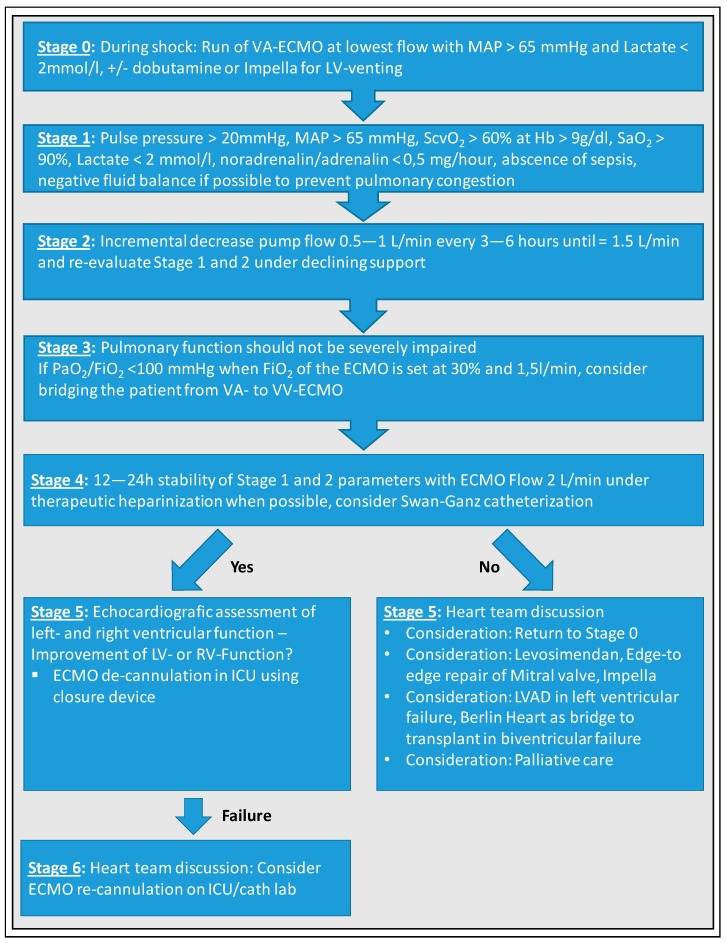
VA-ECMO Weaning—University Hospital Munich, a cardiologic ICU. MAP, mean arterial pressure; ScvO_2_, central venous oxygen saturation; SaO_2_, arterial oxygen saturation; PaO_2_, partial pressure of oxygen; FiO_2_, fraction of inspired oxygen.

As the favored VA-ECMO venting strategy, we use dobutamine as inotropic support for LV unloading to treat increase in afterload due to VA-ECMO support at our center. Furthermore, we run VA-ECMO at the lowest flow rate possible to achieve sufficient perfusion pressure and normal lactate values without Noradrenalin. LV unloading devices like the Impella Cardiac Power (CP) (Abiomed, Danvers, Massachusetts) are implanted in the absence of pulsatility and aortic valve opening as well as presence of relevant pulmonary congestion. We aim for stable pulmonary status with the lowest FiO_2_ levels at respirator and ECMO as possible. A strict negative fluid balance with diuretics whenever possible is aimed to prevent pulmonary congestion while the patient is still under VA-ECMO support. Our weaning strategy roughly adheres to the algorithm of Aissaoui et al. [21] and is displayed in Figure 7.

Weaning trial is intiated, if (1) blood pressure increases, (2) pulsatility of the arterial pressure waveform returns or rises, and (3) echocardiographic parameters assessing LVEF improve. A stepwise reduction of VA-ECMO flow by 0.5 L/min every 3–6 h to a final flow rate around 1.5 L/min is performed. Weaning trial must be performed with sweep gas flow of at least 1 L/min because decreasing sweep gas flow under 1 L/min with FiO_2_ 21% will lead to insignificant oxygenation and decarboxylation with resulting arteriovenous shunt. Patient should be able to maintain at least a mixed venous saturation above 60% (at an Hb value of 9 mg/dL), and an arterial saturation above 90% for 12–24 h. A normal lactate as biomarker is a key requisite during the lowest flow before decannulation. Anticoagulation during weaning attempt is dependent on the presence of bleeding complications as many patients suffer from thrombocytopenia. In many cases, therapeutic heparinization cannot be applied. Once the lowest ECMO flow rate has been reached, at least 12 h of stable hemodynamics is required before final de-cannulation is performed. For de-cannulation itself, we use percutaneous closure devices at bedside in ICU, making resource demanding and stressful transport to the operating room unnecessary [56]. Of course, one important part of every weaning strategy should be the anticipation of failure. New cannulation should be prepared in order to be performed safely and promptly, and revised treatment strategy should be anticipated (e.g., no re-cannulation, LVAD, heart-transplantation, or new attempt few days later).

Although we did not routinely use Swan–Ganz catheters in the past, it might be a promising tool to invasively evaluate LV preload (pulmonary capillary wedge pressure) and RV afterload (mean pulmonary artery pressure) during weaning [57,58]. Issues remain the unreliability of the acquired parameters due to the presence of suction applied by the venous ECMO cannula, which might be negligible at a VA-ECMO flow at 1.5 L/min [5]. RV assessment during venous ECMO flow is also very challenging and might not be reliable at the moment. Here, further analysis is necessary to prove the benefits of these diagnostic tools.

## 9. Conclusions

Patients undergoing VA-ECMO therapy still suffer from extremely high mortality in every day clinical practice. The beneficial effect of VA-ECMO therapy on mortality per se remains unproven and randomized clinical trials have just recently been initiated to address this topic. Weaning strategies are based on institutional standards and individual experiences, but are not covered in former or recent trials. Successful weaning of VA-ECMO is a prerequisite of survival. Different weaning algorithms are discussed and none of them has reached superiority yet. Thus, randomized data are needed but will not be available within the next few years. Therefore, experienced VA-ECMO centers should elaborate standardized weaning algorithms and consensus documents.
